# Supplementary material for: Steroid 21-hydroxylase deficiency dysregulates essential molecular pathways of metabolism and energy provision
Source: Biol Open. 2025 Sep 8;14(9):bio061977. doi: 10.1242/bio.061977 (PMC12452057; doi:10.1242/bio.061977)
Supplement: Supplementary information [file biolopen-14-061977-s1.pdf]

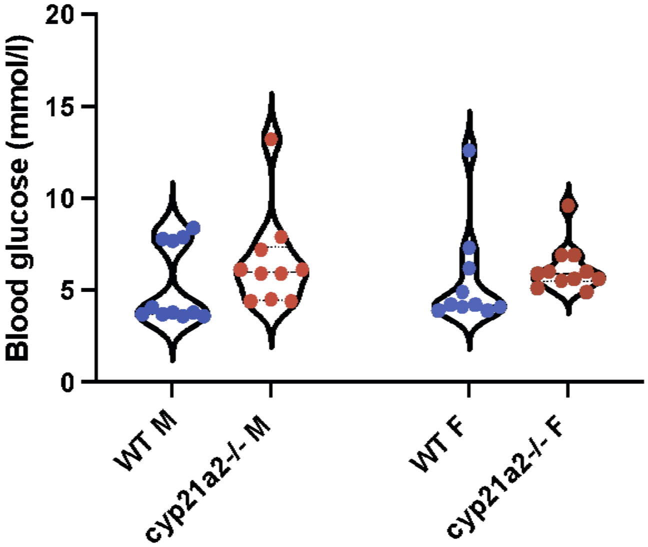

Fig. S1. Blood glucose in zebrafish. Blood glucose concentrations in WT (blue) and mutant (orange) fish of the *cyp21a2* fish. The horizontal lines represent the median with interquartile range. n= 10 - 14 fish.

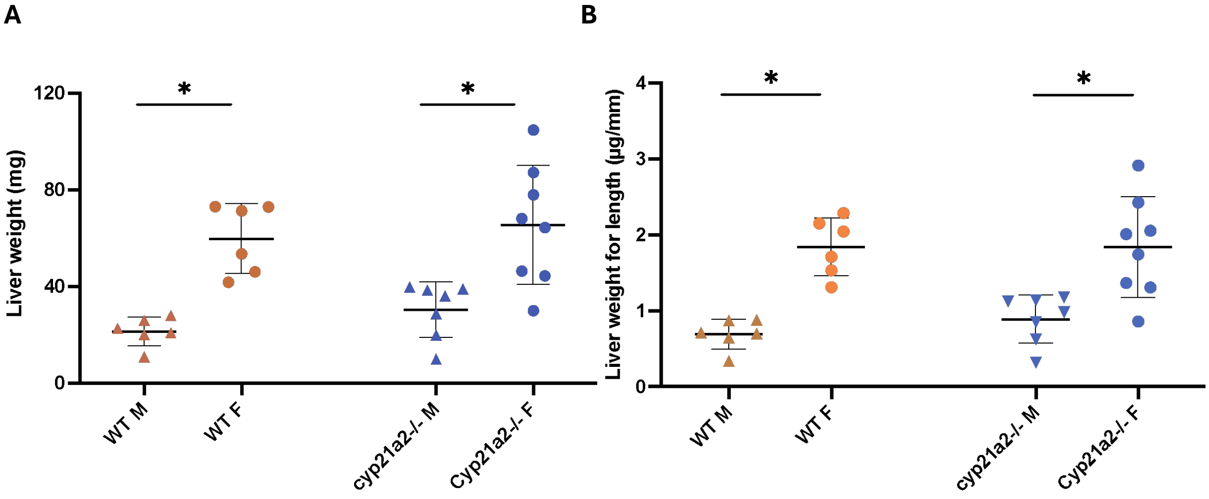

Fig. S2. Liver weight in Cyp21a2-deficient fish

A. Liver weight in WT (orange) and *cyp21a2*-/- (blue), males (M, triangles) and females (F, circles). n= 6 - 8 fish(* = p value < 0.05, unpaired *t*-test). **B.** Liver weight to body length ratio compared between the same groups. The horizontal lines represent the median with interquartile range. n= 6 - 8 fish (* = p value < 0.05, unpaired *t*-test).

Biology Open • Supplementary information

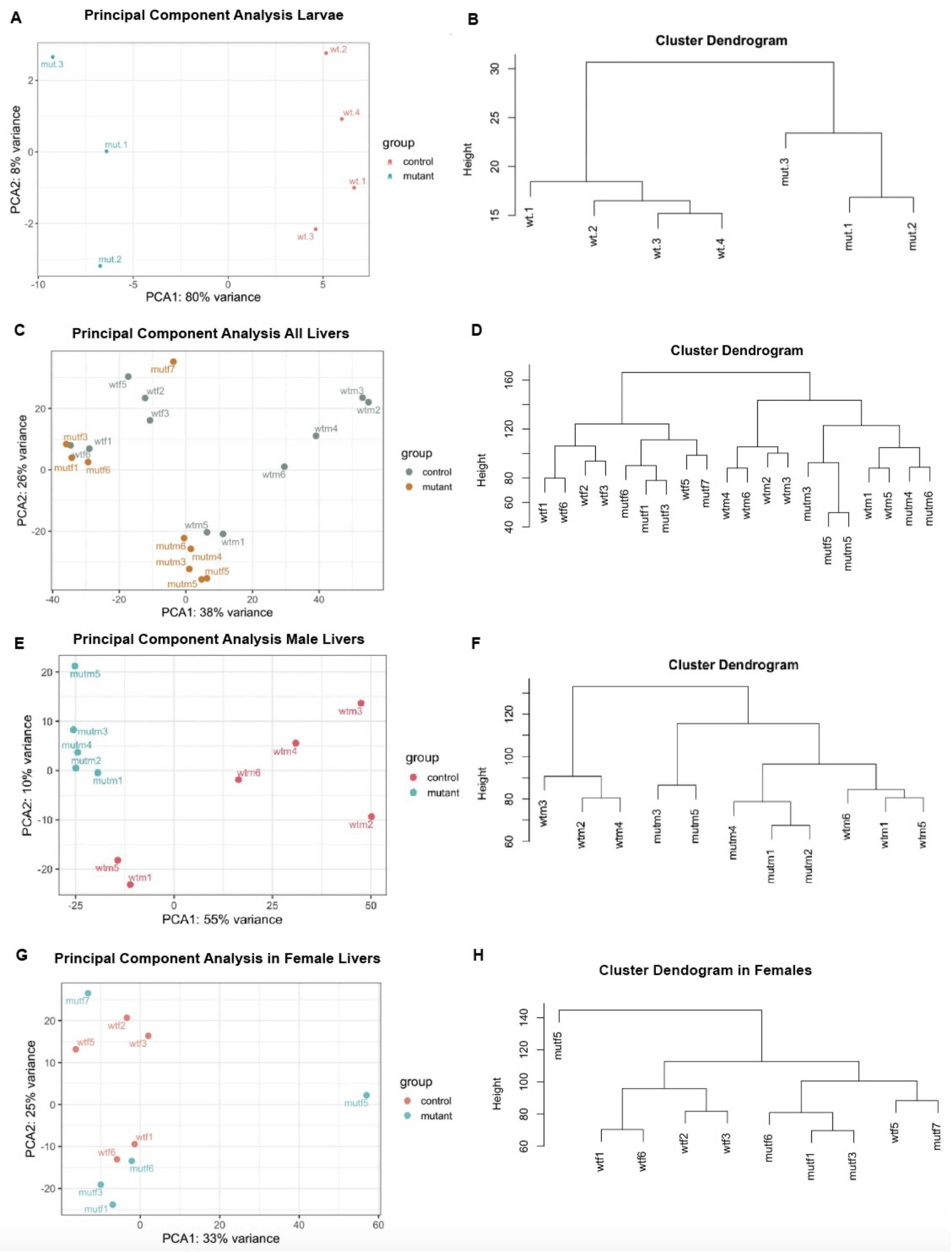

Fig. S3. Differential gene expression caused by the *cyp21a2* mutation in larvae and adult livers. Principal Component Analysis in larvae (**A**), all adult liver samples (**C**), male livers (**E**), and female livers (G); Cluster dendrogram in larvae (B), all adult liver samples (D), male livers (F), and female livers (H).

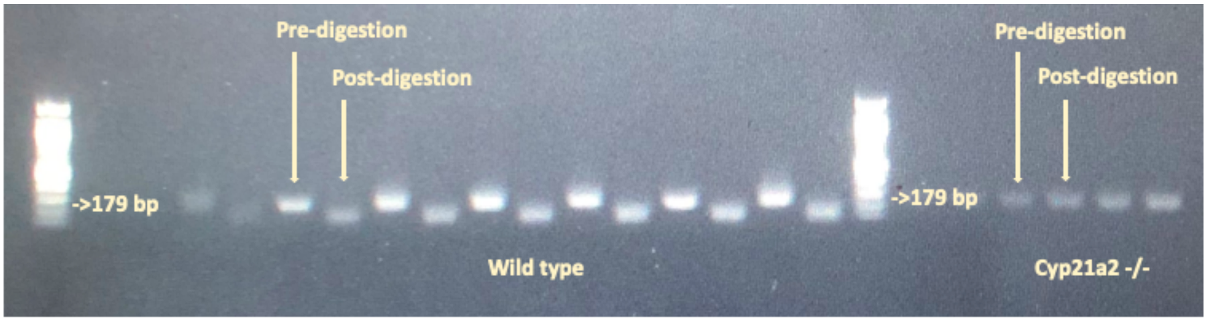

Fig. S4. Genotyping results for the cyp21a2-/- fish, gel electrophoresis. The pairs of bands show the PCR product before and after BseYI digestion. The first row corresponds to wild type fish, showing a smaller DNA fragment after cleavage. The second row corresponds to *cyp21a2*-/- where the cleavage did not take place. The two rows are separated by a DNA ladder.

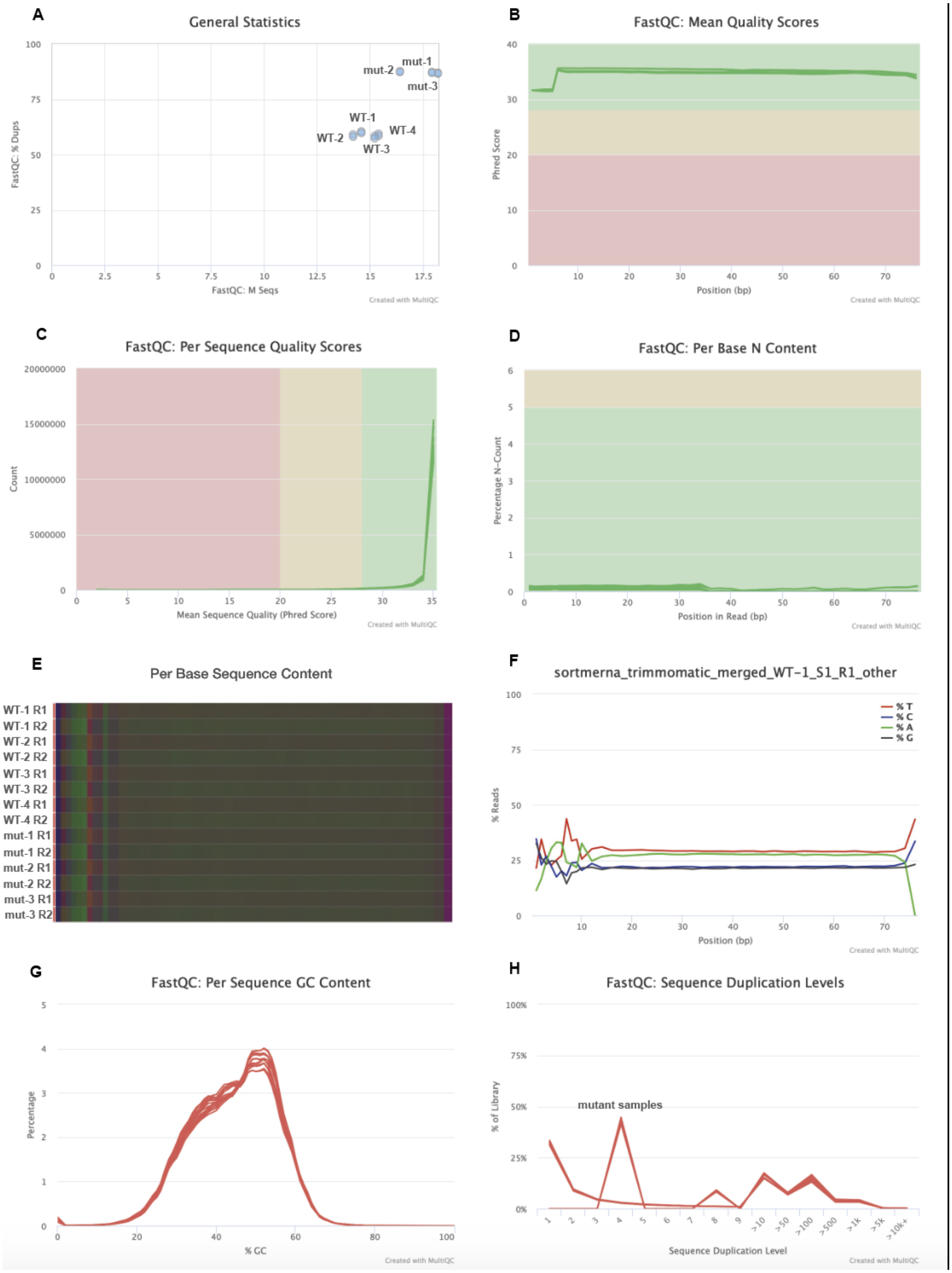

Biology Open • Supplementary information

Fig. S5. Quality of sequencing data in larvae. A. General statistics of the sequencing depth (expressed on the horizontal axis as million reads) plotted against the percentage of sequence duplication (on the vertical axis), showing that mutant samples (mut) had higher sequencing depth and more duplications compared to wild type (WT). **B**. Per base mean quality expressed as the Phred score within the read, showing consistently scores above 30, suggesting high-quality data. **C**. The per sequence quality histogram was shifted to the right towards higher Phred scores. **D**. All samples had very low per base N content (indistinguishable bases). **E**. The heatmap shows all samples failed the per sequence base content; this was due to the bias in the first 12 bases characteristic to Illumina sequencing (**F**), demonstrated in an individual sample (WT-1, R1). **G**. All samples failed the per sequence GC content check due to a consistent skewing to the left of the GC content curve. **H**. All samples failed the sequence duplication level module, with mutant samples presenting 4-level duplication for up to 40% of the library.

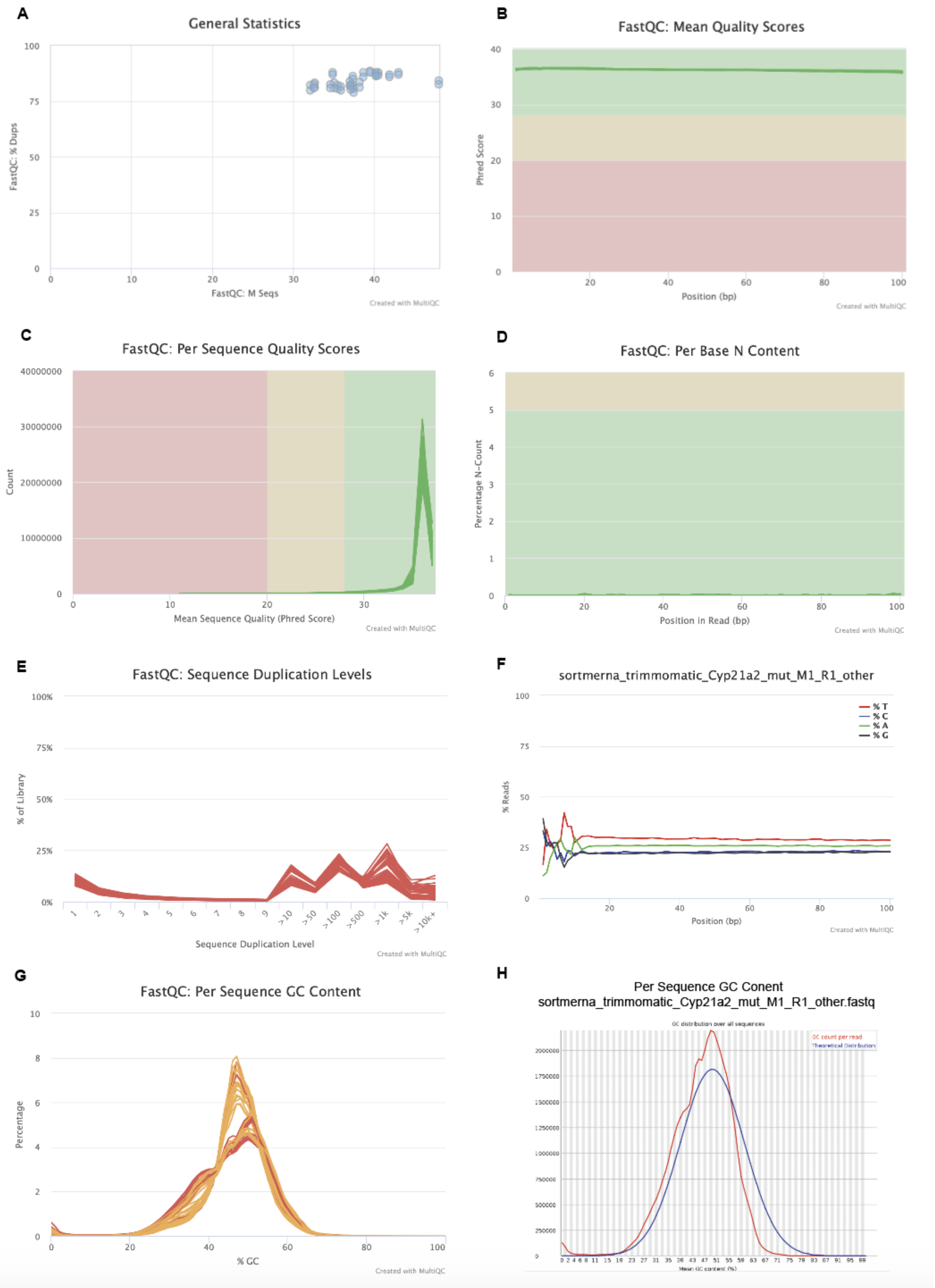

Biology Open • Supplementary information

Fig. 6. Quality of sequencing data in adult livers. A. General statistics of the sequencing depth (expressed on the horizontal axis as million reads) plotted against the percentage of sequence duplication (on the vertical axis). **B**. Per base mean quality expressed as the Phred score within the read, showing consistently scores above 30, suggesting high-quality data. **C**. The per sequence quality histogram was shifted to the right towards higher Phred scores. **D**. All samples had very low per base N content (indistinguishable bases). **E**. All samples failed the sequence duplication level module. **F**. Individual per sequence base content (Mutant Male 1) failed due to the bias in the first 12 bases characteristic to Illumina sequencing. **G**. All samples alarmed or failed the per sequence GC content check due to a consistent skewing to the left of the GC content curve. **H**. Per sequence GC content in an individual sample (Mutant Male 1).

Table S1. Number of mapped reads for each larvae sample

Sample	Wild type 1	Wild type 2	Wild type 3	Wild type 4	Mutant 1	Mutant 2	Mutant 3
Million mapped reads	11.585	11.436	12.199	12.332	14.408	13.199	14.457

(Expressed as million reads)

Table S2. Number of mapped reads for each male liver sample

Sample	Wild type 1	Wild type 2	Wild type 3	Wild type 4	Wild type 5	Wild type 6
Million mapped reads	28.333	31.308	31.855	30.101	28.402	30.060

Biology Open • Supplementary information

Sample	Mutant 1	Mutant 2	Mutant 3	Mutant 4	Mutant 5
Million mapped reads	41.1833	32.726	30.536	31.788	30.230

(Expressed as million reads)

Gene ontology enrichment analysis

Table 3. Top 10 biological process identified by GO enrichment analysis in GOrilla for cyp21a2-/- larvae

GO Term	Description	Enrichment (N,B,n,b)	p-value	FDR q-value
GO:0044281	Small molecule metabolic process	1.7 (14317, 920, 1205, 132)	6.58e-09	5.65e-05
GO:0006082	Organic acid metabolic process	1.95 (14317, 506, 1205, 83)	2.44e-08	1.05e-04
GO:1903047	Mitotic cell cycle process	2.35 (14317, 217, 1429, 51)	2.87e-08	8.20e-05
GO:0019752	Carboxylic acid metabolic process	2 (14317, 445, 1205, 75)	4.03e-08	8.64e-05
GO:0022402	Cell cycle process	2.02	1.76e-07	3.02e-04

Biology Open • Supplementary information

		(14317, 338, 1387, 66)		
GO:0043436	Oxoacid metabolic process	1.93<br><br>(14317, 469, 1205, 76)	1.78e-07	2.55e-04
GO:0017144	Drug metabolic process	2.27<br><br>(14317, 276, 1074, 47)	6.62e-07	8.12e-04
GO:0051726	Regulation of cell cycle	2.19<br><br>(14317, 293, 1115, 50)	7.99e-07	8.57e-04
GO:0009056	Catabolic process	2.01<br><br>(14317, 962, 444, 60)	1.98e-06	1.89e-03
GO:1901605	Alpha-amino acid metabolic process	2.61<br><br>(14317, 141, 1205, 31)	2.67e-06	2.29e-03

The **p-value** is the enrichment p-value computed according to the mHG or HG model. The **FDR q-value** is the correction of the p-value for multiple testing using the Benjamini and Hochberg (1995) method. The **Enrichment = (b/n) / (B/N) (**N - total number of genes, B - total number of genes associated with a specific GO term, n – a flexible cut-off, being an automatically determined number of genes in the input list or 'target set', b - is the number of genes in the target set that are associated with the GO term).

Table S4. Top 10 biological process identified by GO enrichment analysis in GOrilla for *cyp21a2* male livers

GO Term	Description	Enrichment<br><br>(N,B,n,b)	p-value	FDR<br><br>q-value
GO:0015711	Organic anion transport	2.75<br><br>(13643, 160, 1551, 50)	7.28e-11	6.21e-07
GO:0006820	Anion transport	2.34	2.16e-10	9.23e-07

Biology Open • Supplementary information

		(13643, 244, 1551, 65)		
GO:0008202	Steroid metabolic process	7.16<br><br>(13643, 88, 368, 17)	2.73e-09	7.78e-06
GO:0046942	Carboxylic acid transport	3.07<br><br>(13643, 103, 1551, 36)	4.42e-09	9.43e-06
GO:0015849	Organic acid transport	3.02<br><br>(13643, 105, 1551, 36)	8.07e-09	1.38e-05
GO:0006629	Lipid metabolic process	2.59<br><br>(13643, 547, 327, 34)	3.64e-06	5.17e-03
GO:0033555	Multicellular organism response to stress	1,240.27<br><br>(13643, 11, 2, 2)	3.79e-06	4.63e-03
GO:0044281	Small molecule metabolic process	1.56<br><br>(13643, 891, 1175, 120)	4.46e-06	4.75e-03
GO:0006811	Ion transport	1.94<br><br>(13643, 676, 624, 60)	6.42e-06	6.08e-03
GO:1901615	Organic hydroxy compound metab. proc.	3.77<br><br>(13643, 172, 379, 18)	7.66e-06	6.54e-03

Biological Process GO term analysis of genes exhibiting differential expression in adult male WT and *cyp21a2-/-* mutant livers, using GOrilla. The **p-value** is the enrichment p-value computed according to the mHG or HG model. The **FDR q-value** is the correction of the p-value for multiple testing using the Benjamini and Hochberg (1995) method. The **Enrichment = (b/n) / (B/N) (**N - total number of genes, B - total number of genes associated with a specific GO term, n – a flexible cut-off, being an automatically determined number of genes in the input list or ‘target set’, b - is the number of genes in the target set that are associated with the GO term).

Biology Open • Supplementary information

Table S5. Top 10 biological process identified by GO enrichment analysis in GOrilla for *cyp21a2* female livers

GO Term	Description	Enrichment (N,B,n,b)	p-value	FDR q-value
GO:0034470	ncRNA processing	5.87 (13653, 227, 1466, 143)	3.93E-82	3.35E-78
GO:0034660	ncRNA metabolic process	5.1 (13653, 281, 1466, 154)	3.93E-76	1.68E-72
GO:0016070	RNA metabolic process	3.1 (13653, 730, 1466, 243)	7.75E-65	2.21E-61
GO:0006396	RNA processing	3.67 (13653, 482, 1466, 190)	1.35E-63	2.88E-60
GO:0006364	rRNA processing	6.95 (13653, 122, 1466, 91)	1.69E-62	2.89E-59
GO:0016072	rRNA metabolic process	6.6 (13653, 134, 1466, 95)	8.21E-62	1.17E-58
GO:0034641	cellular nitrogen compound metabolic process	2.15 (13653, 1768, 1466, 409)	6.82E-60	8.32E-57
GO:0090304	nucleic acid metabolic process	2.53 (13653, 1034, 1466, 281)	3.00E-54	3.20E-51

Biology Open • Supplementary information

GO:0006139	nucleobase-containing compound metabolic process	2.23 (13653, 1348, 1466, 323)	2.00E-49	1.90E-46
GO:1901360	organic cyclic compound metabolic process	2.1 (13653, 1538, 1466, 346)	1.70E-46	1.32E-43

Biological Process GO term analysis of genes exhibiting differential expression in adult female WT and *cyp21a2-/-* mutant livers, using GOrilla. The **p-value** is the enrichment p-value computed according to the mHG or HG model. The **FDR q-value** is the correction of the p-value for multiple testing using the Benjamini and Hochberg (1995) method. The **Enrichment = (b/n) / (B/N) (**N - total number of genes, B - total number of genes associated with a specific GO term, n – a flexible cut-off, being an automatically determined number of genes in the input list or 'target set', b - is the number of genes in the target set that are associated with the GO term).

Table S6. Associations between the cyp21a2-/- female liver transcriptome and human disease (top 20 conditions)

Description	p-value	FDR q-value
Metabolic Syndrome X	1.83E-18	1.62E-14
Non-alcoholic Fatty Liver Disease	3.92E-17	1.73E-13
Obesity	7.01E-16	2.07E-12
Neutropenia	2.30E-15	4.06E-12
Colon Carcinoma	2.21E-15	4.06E-12
Leukopenia	3.01E-15	4.44E-12
Mammary Neoplasms	4.64E-15	5.86E-12

Biology Open • Supplementary information

Malignant tumor of colon	6.57E-15	7.26E-12
Diabetes Mellitus, Non-Insulin-Dependent	1.92E-14	1.41E-11
Lucey-Driscoll syndrome (disorder)	1.90E-14	1.41E-11
Crigler Najjar syndrome, type 2	1.90E-14	1.41E-11
Carcinoma	2.84E-14	1.93E-11
Dyslipidemias	1.70E-13	1.07E-10
Rheumatoid Arthritis	7.99E-13	4.71E-10
Hypertensive disease	1.89E-12	1.04E-09
Secondary Neoplasm	3.33E-12	1.73E-09
Hypertriglyceridemia	3.75E-12	1.78E-09
Autoimmune hepatitis type 2	3.83E-12	1.78E-09
Hepatitis C	5.49E-12	2.31E-09
Liver Cirrhosis, Experimental	5.47E-12	2.31E-09

The p-value is the enrichment p-value computed according to the mHG or HG model. The FDR q-value is the correction of the p-value for multiple testing using the Benjamini and Hochberg (1995) method.

Table S7.

Available for download at
https://journals.biologists.com/bio/article-lookup/doi/10.1242/bio.061977#supplementary-data
